# Structural and dynamic changes associated with beneficial engineered single-amino-acid deletion mutations in enhanced green fluorescent protein

**DOI:** 10.1107/S139900471401267X

**Published:** 2014-07-25

**Authors:** James A. J. Arpino, Pierre J. Rizkallah, D. Dafydd Jones

**Affiliations:** a School of Biosciences, Cardiff University, Park Place, Cardiff CF10 3AT, Wales; b School of Medicine, Cardiff University, Heath Park, Cardiff CF14 4XN, Wales

**Keywords:** enhanced green fluorescent protein, single-amino-acid deletions, protein engineering

## Abstract

Single-amino-acid deletions are a common part of the natural evolutionary landscape but are rarely sampled during protein engineering owing to limited and prejudiced molecular understanding of mutations that shorten the protein backbone. Single-amino-acid deletion variants of enhanced green fluorescent protein (EGFP) have been identified by directed evolution with the beneficial effect of imparting increased cellular fluorescence. Biophysical characterization revealed that increased functional protein production and not changes to the fluorescence parameters was the mechanism that was likely to be responsible. The structure EGFP^D190Δ^ containing a deletion within a loop revealed propagated changes only after the deleted residue. The structure of EGFP^A227Δ^ revealed that a ‘flipping’ mechanism was used to adjust for residue deletion at the end of a β-strand, with amino acids C-terminal to the deletion site repositioning to take the place of the deleted amino acid. In both variants new networks of short-range and long-range interactions are generated while maintaining the integrity of the hydrophobic core. Both deletion variants also displayed significant local and long-range changes in dynamics, as evident by changes in *B* factors compared with EGFP. Rather than being detrimental, deletion mutations can introduce beneficial structural effects through altering core protein properties, folding and dynamics, as well as function.

## Abstract 2

The beneficial engineered single-amino-acid deletion variants EGFP^D190Δ^ and EGFP^A227Δ^ have been studied.

## 1. Introduction   

**Figure 1:**
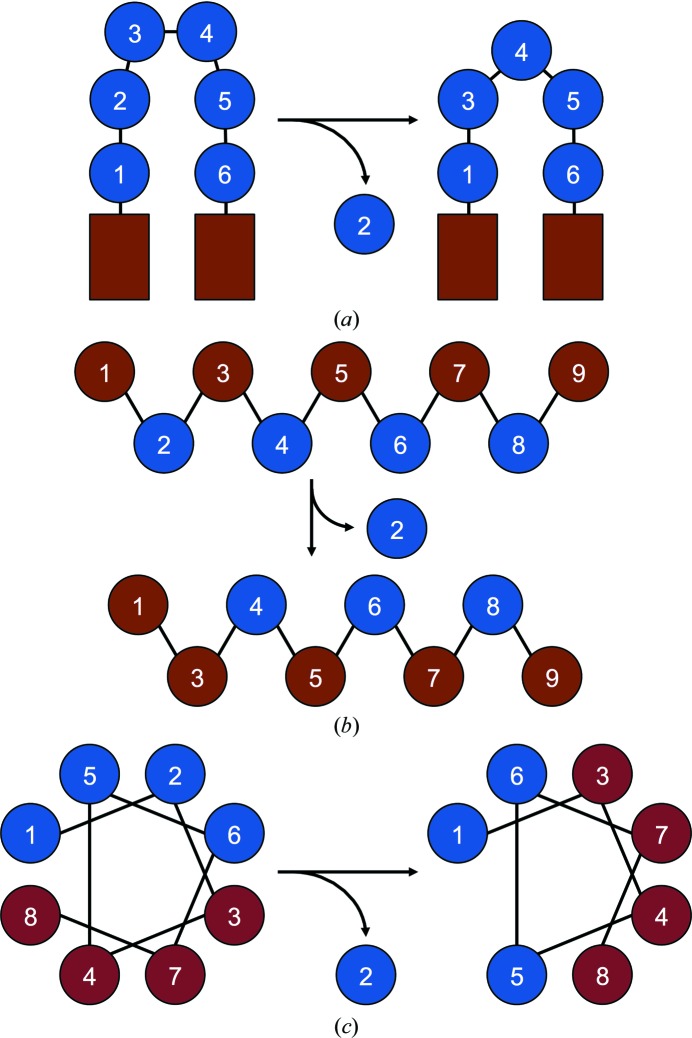
Effect of single-amino-acid deletions on secondary-structure registry. (*a*) Deletion of a single amino acid (blue circle) from a loop region connecting two ordered secondary-structural elements (red rectangles) is usually accommodated by loop shortening. Deletion of an amino acid from (*b*) a β-strand or (*c*) an α-helix results in registry shifts. Amino acids are coloured red or blue to distinguish between different faces of a secondary structure.

Targeted gene mutagenesis has revolutionized our ability to interact with and engineer proteins for both fundamental studies of the folding–structure–function relationship (Brannigan & Wilkinson, 2002 ▶) and technological use (Channon *et al.*, 2008 ▶; Cherry & Fidantsef, 2003 ▶). Whether rational site-directed mutagenesis, computational design or library-based directed-evolution approaches are used, the focus is the generation of amino-acid substitutions (Goldsmith & Tawfik, 2012 ▶; Tracewell & Arnold, 2009 ▶). The natural evolutionary process, which one can argue is the most successful protein-engineering algorithm, goes beyond utilizing substitution mutations alone, sampling amino-acid insertion and deletion (InDel) events. InDels are distinct from substitutions as they affect the polypeptide backbone and not just the side chain (Chothia *et al.*, 2003 ▶; de Jong & Rydén, 1981 ▶; Taylor *et al.*, 2004 ▶; Wang *et al.*, 2009 ▶; Tóth-Petróczy & Tawfik, 2013 ▶; Leushkin *et al.*, 2012 ▶). Despite InDel mutations sampling distinct sequence space and hence structural events (Pascarella & Argos, 1992 ▶; Shortle & Sondek, 1995 ▶), they are generally ignored as part of normal protein-engineering endeavours. This is in part owing to the difficulty in predicting the local and global structural rearrangements on altering the protein backbone, despite some recent insights (Arpino, Czapinska *et al.*, 2012 ▶; Heinz *et al.*, 1993 ▶; O’Neil *et al.*, 2000 ▶; Simm *et al.*, 2007 ▶; Edwards *et al.*, 2010 ▶; Vetter *et al.*, 1996 ▶; Stott *et al.*, 2009 ▶; Jones *et al.*, 2000 ▶; Jones & Perham, 2008 ▶). Dogma also suggests that InDels are likely to be detrimental to proteins owing to, for example, registry shifts in organized secondary structure (Pascarella & Argos, 1992 ▶). These assumptions are based largely on simple models of the structural impact of InDels (see Fig. 1 ▶). As a result, the impact of such an important class of mutations on the protein folding–structure–function relationship has not been widely explored in terms of both their fundamental molecular mechanism of action and their technological application. This is despite recent evidence that InDels can be a key driver of major leaps in protein fitness through adaptation of function and structure during evolution, and thus have a role to play in protein engineering (Leushkin *et al.*, 2012 ▶; Tóth-Petróczy & Tawfik, 2013 ▶).

Amongst the InDel events observed, which range from single-nucleotide deletions to the insertion of whole domains, single amino-acid deletions (*via* the removal of a contiguous trinucleotide sequence) are one of the most commonly observed amongst functional protein homologues (deJong & Rydén, 1981 ▶; Taylor *et al.*, 2004 ▶; Pascarella & Argos, 1992 ▶; Tóth-Petróczy & Tawfik, 2013 ▶; Leushkin *et al.*, 2012 ▶). From a protein-engineering perspective, deletion mutations may be considered to be more harmful than their insertional counterparts as the protein backbone is becoming more constrained; insertions can be tolerated through expansion of segments or ‘looping out’. However, there are a growing number of examples that show that deletion mutations can have beneficial effects (Afriat-Jurnou *et al.*, 2012 ▶; de Wildt *et al.*, 1999 ▶; Simm *et al.*, 2007 ▶; Wood *et al.*, 2009 ▶). For example, various single-amino-acid deletion variants of TEM β-lactamase have been identified that improved activity towards normally poor β-lactam substrates (Simm *et al.*, 2007 ▶).

The recent advent of directed-evolution approaches to sample InDel mutations across a protein backbone without any perceived prejudice (Fujii *et al.*, 2006 ▶; Jones, 2005 ▶; Murakami *et al.*, 2002 ▶; Edwards *et al.*, 2008 ▶; Guntas *et al.*, 2004 ▶) has provided a route to gain information on the general tolerance and structure–function effects of deletion mutations. These approaches rely on the removal or insertion of contiguous nucleotide segments at random positions in a target gene. In particular, the use of an engineered version of the Mu transposon (termed MuDel) with low insertion-site specificity (Edwards *et al.*, 2008 ▶; Baldwin *et al.*, 2008 ▶, 2009 ▶; Jones, 2005 ▶) allows the removal of a single contiguous trinucleotide sequence per gene (Jones, 2005 ▶). Application of this approach has resulted in one of the most detailed surveys to date concerning the general tolerance of the commonly used enhanced version of the *Aequorea victoria* green fluorescent protein (EGFP; Tsien, 1998 ▶; Ormö *et al.*, 1996 ▶; Yang *et al.*, 1996 ▶; Arpino, Czapinska *et al.*, 2012 ▶; Royant & Noirclerc-Savoye, 2011 ▶) to single-amino-acid deletions (Arpino *et al.*, 2014 ▶). One variant with Gly4 in an N-terminal 3_10_-helix deleted conferred a much brighter fluorescence phenotype on *Escherichia coli*. Structural analysis revealed that more efficient folding through the formation of new long-range polar interactions, including to the sole *cis*-proline bond between Met88 and Pro89, was responsible. Here, we report the detailed structural and functional characterization of two additional variants isolated from the EGFP single-amino-acid deletion library. Both variants confer increased fluorescence brightness on *E. coli* and exert their influence through propagated interactions that alter both local and long-range bond networks and dynamics, features that are common to all proteins, rather than changes to intrinsic function.

## 2. Methods and materials   

### 2.1. Protein production and purification   

The EGFP deletion variants were isolated from a tri­nucleotide deletion library as described previously (Arpino *et al.*, 2014 ▶). The production and subsequent purification of the proteins was performed essentially as described previously (Arpino, Rizkallah *et al.*, 2012 ▶; Arpino *et al.*, 2014 ▶). The production of EGFP, EGFP^D190Δ^ and EGFP^A227Δ^ for whole-cell fluorescence analysis was performed as follows. LB Broth (20 ml) supplemented with 100 µg ml^−1^ ampicillin and 1 m*M* IPTG was inoculated with a single *E. coli* BL21-Gold (DE3) colony containing the relevant plasmid (pNOM-XP3 containing the *egfp*, *egfp^D190Δ^ or egfp^A227Δ^* genes) and incubated overnight at 37°C.

### 2.2. Fluorescence spectroscopy   

All fluorescence studies were performed using a Cary Eclipse fluorescence spectrophotometer (Varian). Excitation and emission spectra were measured in a cuvette of dimensions 5 × 5 mm with a 10 nm excitation and emission band pass at a scan rate of 600 nm min^−1^. Excitation scans were measured by monitoring emission at 511 nm and emission was measured after excitation at 488 nm. Whole-cell fluorescence spectroscopy was performed on *E. coli* BL21-Gold (DE3) cell cultures after expression of EGFP or single-amino-acid deletion variants of EGFP. Expression cultures were harvested by centrifugation (1500*g* for 10 min) and all supernatant was removed and discarded. The cell pellet was resuspended in 50 m*M* Tris–HCl pH 8.0 at 25°C, 150 m*M* NaCl, 10%(*v*/*v*) glycerol (TNG buffer) to an OD_600_ of 0.1 in a 1 cm path-length cuvette. The resuspended cells were transferred into a cuvette of dimensions 5 × 5 mm and excitation and emission spectra were measured as described above. Calculation of quantum yield and fluorescence lifetimes were performed as described previously (Arpino, Czapinska *et al.*, 2012 ▶; Arpino, Rizkallah *et al.*, 2012 ▶).

### 2.3. Protein crystallization and structure determination   

Purified EGFP^D190Δ^ and EGFP^A227Δ^ (15 mg ml^−1^ in 50 m*M* Tris–HCl pH 8.0, 150 m*M* NaCl) were screened for crystal formation by the sitting-drop vapour-diffusion method with incubation at 18°C. Drops were set up with equal volumes of protein and precipitant solution (0.5 µl each). Crystals of EGFP^D190Δ^ were obtained from 0.1 *M* sodium cacodylate pH 6.5, 0.2 *M* NaCl, 1 *M* sodium citrate. Mother liquor (0.5 µl) supplemented with 15–25%(*v*/*v*) ethylene glycol was added to the crystal-containing drops as a cryoprotectant and crystals were mounted and vitrified in liquid nitrogen. Crystals of EGFP^A227Δ^ were obtained from 0.1 *M* MMT buffer (malic acid, MES and Tris) pH 4.0, 25%(*w*/*v*) PEG 1500. Crystals were mounted directly from mother liquor with no cryoprotectant and were vitrified. Data were collected on beamlines I03 (EGFP^D190Δ^) or I04 (EGFP^A227Δ^) at the Diamond Light Source.

Data were reduced with the *xia*2 package (Winter, 2009 ▶), space-group assignment was performed by *POINTLESS* (Evans, 2006 ▶) and scaling and merging were completed with *SCALA* (Evans, 2006 ▶) and *TRUNCATE* from *CCP*4 (Winn *et al.*, 2011 ▶). Initial molecular replacement for the EGFP deletion-variant structures was performed using a previously determined EGFP structure (PDB entry 4eul; Arpino, Rizkallah *et al.*, 2012 ▶) as the search model using *Phaser* (McCoy *et al.*, 2007 ▶). The structures of the EGFP deletion variants were adjusted manually using *Coot* (Emsley *et al.*, 2010 ▶) and refinement of the completed molecule was carried out using *REFMAC* (Murshudov *et al.*, 2011 ▶). Protein atoms were refined isotropically and anisotropically. All nonprotein atoms were refined isotropically. The above routines were used within the *CCP*4 package (Winn *et al.*, 2011 ▶; http://www.ccp4.ac.uk). Graphical representations were generated with *PyMOL* (Schrödinger).

## 3. Results and discussion   

### 3.1. Single-amino-acid deletions   

The role of InDel mutations, including single-amino-acid deletions, in shaping the modern protein repertoire is clear (Leushkin *et al.*, 2012 ▶; Tóth-Petróczy & Tawfik, 2013 ▶; Pascarella & Argos, 1992 ▶). However, given the structural changes required to accommodate an amino-acid removal and the subsequent influence on the connected interactions (both directly connected to the amino acid removed and the rearranged adjacent residues), predicting the effects of a single-amino-acid deletion is currently difficult compared with that of a substitution. It is difficult to ascertain the impact of a deletion alone through analysis of structural homologues, as additional mutations may have modulated the original molecular events. Therefore, there is a need to acquire detailed experimental information on the sole structural consequences of amino-acid deletions. This has only been exemplified to a limited extent, for example, in T4 lysozyme (Vetter *et al.*, 1996 ▶), the B-domain of protein G (O’Neil *et al.*, 2000 ▶) and lipoyl domains (Jones *et al.*, 2000 ▶; Jones & Perham, 2008 ▶; Stott *et al.*, 2009 ▶).

In terms of the general effect of an amino-acid deletion on structure, three coarse models are normally proposed (Fig. 1 ▶) depending on the type of secondary structure. Historical analysis of protein homologues suggests that deletions of short stretches of amino acids from loops are generally considered to be the most tolerant owing to the increased conformational flexibility and heterogeneity in these regions of a protein (Pascarella & Argos, 1992 ▶); the mutation is accommodated through simple loop shortening (Fig. 1 ▶ *a*). Deletions of an amino acid from the middle of a β-strand are considered to be detrimental as they could cause the local rearrangement of amino acids in the regularly ordered strand, resulting in a shift of the side chains from one face of the β-strand to the other (Fig. 1 ▶ *b*) and potentially having knock-on effects on the global structure (O’Neil *et al.*, 2000 ▶). For example, if the side chains on one face of a surface-exposed β-strand were predominantly polar and those on the opposite face were predominantly hydrophobic and buried in the core of the protein, an amino-acid deletion may cause a register shift and reverse the environment sampled by each amino acid. The result could be hydrophobic residues becoming solvent-exposed and the polar side chains being buried into the core of the protein (Fig. 1 ▶ *b*). A similar effect may occur if an amino acid were to be deleted from a helix, with the potential result being a rotation of all of the side-chain positions around the α-helix (Fig. 1 ▶ *c*). However, unlike helices, β-strands are rarely stand-alone elements but form part of a networked β-sheet structure, so the implications of disrupting a single strand may be more widespread. As well as affecting registry in organized secondary structures, amino-acid deletion could also result in the shortening of secondary structure and adjacent loop expansion. This may favour deletions occurring towards the termini of secondary-structure elements (Pascarella & Argos, 1992 ▶; Vetter *et al.*, 1996 ▶); there is some evidence that the latter effect is occurring in EGFP (Arpino *et al.*, 2014 ▶). Here, we present the structural and functional analysis of two variants of EGFP that sample a deletion in a β-strand (EGFP^A227Δ^) or in a loop (EGFP^D190Δ^). Together with the previously reported variant EGFP^G4Δ^ (Arpino *et al.*, 2014 ▶) containing a deletion in a helical segment, we aim to advance our knowledge relating to the structural description of the beneficial impact of deletion residues within each of the main secondary-structure elements within a single protein scaffold.

### 3.2. Fluorescence properties of EGFP^D190Δ^ and EGFP^A227Δ^   

**Figure 2:**
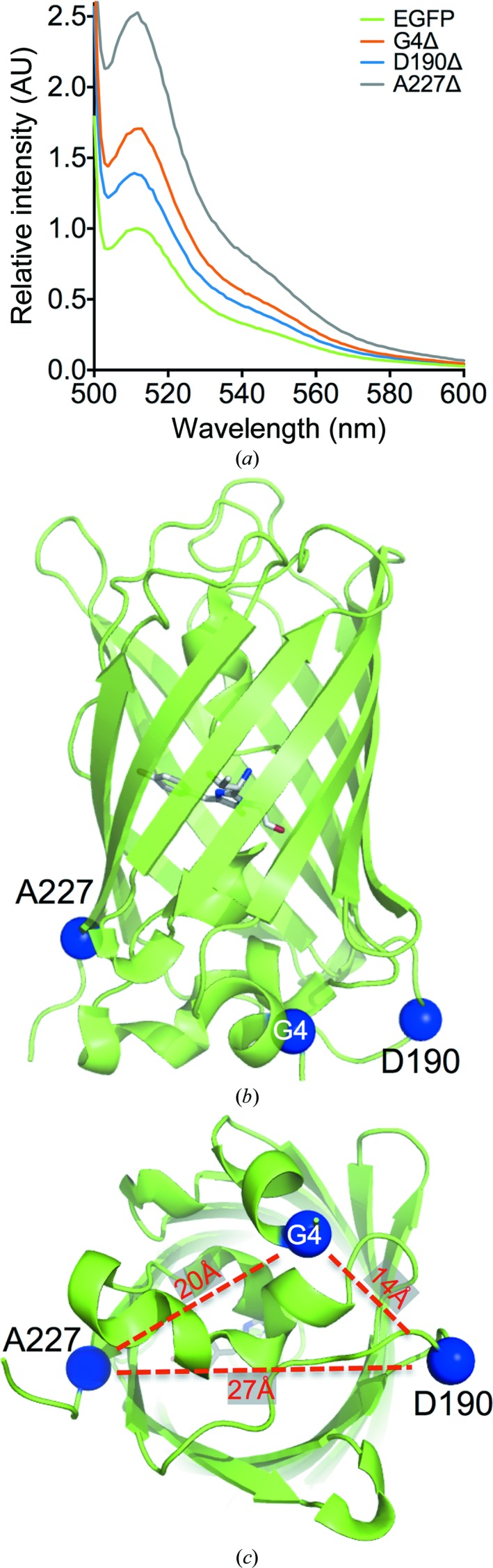
Whole-cell fluorescence spectra and single-amino-acid deletion positions. (*a*) Whole-cell fluorescence spectra normalized to the EGFP emission maxima. (*b*) Side and (*c*) bottom views of the tertiary structure of EGFP (PDB entry 4eul) with the chromophore shown as sticks and single-amino-acid deletion positions highlighted by blue spheres. In (*c*), the distances between the residues are shown.

EGFP has proved to be an important tool in cell biology. It is one of the most widely used versions of auto-fluorescent proteins based on the original *A. victoria* GFP (Tsien, 1998 ▶) and an important target for protein engineering. EGFP is an archetypical autofluorescent protein in terms of structure and function (Fig. 2 ▶; Arpino, Rizkallah *et al.*, 2012 ▶; Royant & Noirclerc-Savoye, 2011 ▶; Tsien, 1998 ▶). It comprises a core β-barrel capped at each end. Running through the centre of the barrel is a kinked helix that houses the distinctive *p*-hydroxybenzylidene-imidazolinone (HBI) chromophore. HBI forms as a result of covalent rearrangement of three residues resident in the central helix (Thr65-Tyr67-Gly68). Fluorescence is linked and modulated by the interaction of HBI with other residues buried within the barrel. GFP has been the focus of previous InDel-based protein-engineering approaches (Flores-Ramírez *et al.*, 2007 ▶; Li *et al.*, 1997 ▶; Dopf & Horiagon, 1996 ▶), including domain insertion (Arpino, Czapinska *et al.*, 2012 ▶; Baird *et al.*, 1999 ▶; Doi & Yanagawa, 1999 ▶; Biondi *et al.*, 1998 ▶; Nakai *et al.*, 2001 ▶), but these have generally been focused on targeted regions.

Using a transposon-based trinucleotide-deletion (TND) approach (Jones, 2005 ▶; Simm *et al.*, 2007 ▶), a library of single-amino-acid deletions across the breadth of EGFP was constructed as reported previously (Baldwin *et al.*, 2008 ▶; Arpino *et al.*, 2014 ▶). The study revealed that the loops and helices that lie at either end of the core barrel along with the termini of β-strands are most tolerant to amino-acid deletion; the middle of strands that comprise the β-barrel and residues with low solvent exposure are less tolerant. Screening of the library after transformation of *E. coli* revealed that on irradiation certain colonies appeared brighter than the general background level. This observation was confirmed by whole-cell fluorescence spectroscopy (Fig. 2 ▶ *a*). Sequencing of the EGFP genes from these colonies revealed that three deletion mutations dominated: G4Δ, D190Δ and A227Δ (Fig. 2 ▶ *b*). Each of the three variants are present in different secondary-structure elements and have different solvent accessibility, but all reside close to each other at one end of the β-barrel that is thought to comprise a lid during the later stages of GFP folding (Fig. 2 ▶ *c*; Andrews *et al.*, 2008 ▶). EGFP^G4Δ^ that has Gly4 deleted in the N-terminal H1 helix has been described previously (Arpino *et al.*, 2014 ▶). Removal of Asp190 in a ten-residue loop linking β-strands S9 and S10 results in a ∼1.4-fold higher whole-cell fluorescence compared with EGFP (Fig. 2 ▶ *a*). The EGFP^A227Δ^ variant conferred the brightest phenotype on *E. coli* grown at 37°C, with a 2.6-fold increase in cellular fluorescence compared with EGFP (Fig. 2 ▶ *a*). Ala227 resides at the end of the final β-strand comprising the β-barrel of EGFP (Fig. 2 ▶ *b*). Both Gly4 and Ala227 are relatively buried, with a solvent-accessible surface area (SASA) of 2.8 and 28.6 Å^2^ (backbone, 3.7 Å^2^), respectively. This is contrary to the general trend, in which surface-exposed residues are more likely to be tolerant to a single-amino-acid deletion in EGFP (Arpino *et al.*, 2014 ▶). Asp190 is essentially completely exposed to the solvent, with a SASA of 152.8 Å^2^ (backbone, 31 Å^2^).

There does appear to be a strict context concerning the beneficial effects of the three identified deletions. As reported previously, in the context of helix H1 and the N-terminal region only deletion of Gly4 exerts a beneficial effect; removal of Glu5 and Glu6 does not improve cellular brightness to a great extent and removal of Lys3 in combination with a G4S substitution mutation was not tolerated (Arpino *et al.*, 2014 ▶). With respect to Asp190, removal of the adjacent Gly189 or the close-by Pro192 reduces the apparent cellular fluorescence; deletion of Pro187 renders the protein nonfluorescent (Arpino *et al.*, 2014 ▶). The same is true with regard to Ala227. Deletion of Gly228 is tolerated but reduces the apparent cellular fluorescence by approximately fivefold. Deletion of Ala227 together with Ala226 was also observed (A226Δ-A227Δ) and was tolerated by EGFP. A slightly improved apparent cellular fluorescence was observed for this variant but not to the same extent as A227Δ alone (Supplementary Fig. S1^1^).

To understand the basis for the improved cellular fluorescence observed for EGFP^G4Δ^, EGFP^D190Δ^ and EGFP^A227Δ^, a more detailed *in vitro* analysis of the purified proteins was undertaken. The data for EGFP^G4Δ^ have been reported elsewhere (Arpino *et al.*, 2014 ▶), but are included here for comparison. The fluorescence parameters of each variant were essentially similar to those of EGFP (Table 1 ▶). The quantum yields and molar extinction coefficients were essentially identical to those of EGFP, resulting in each variant having a similar brightness. This suggests that the mutations are not affecting the fluorescence properties *per se*, but that increased brightness is a result of more efficient production of correctly folded fluorescing protein in the cell.

### 3.3. Structural impact of D190Δ and A227Δ mutations on EGFP   

To understand the structural impact that the deletion mutations have on EGFP, the three deletion variants EGFP^G4Δ^, EGFP^D190Δ^ and EGFP^A227Δ^ were crystallized. The structures of both EGFP (to 1.35 and 1.50 Å resolution; PDB entries 4eul and 2yog; Arpino, Rizkallah *et al.*, 2012 ▶; Royant & Noirclerc-Savoye, 2011 ▶) and EGFP^G4Δ^ (to 1.6 Å resolution; PDB entry 4ka9; Arpino *et al.*, 2014 ▶) have been determined previously. Size-exclusion chromatography suggested that like EGFP (Arpino, Rizkallah *et al.*, 2012 ▶) and EGFP^G4Δ^ (Arpino *et al.*, 2014 ▶), EGFP^D190Δ^ and EGFP^A227Δ^ were essentially monomeric (Supplementary Fig. S2). EGFP^D190Δ^ and EGFP^A227Δ^ were crystallized in their native sequence form (residues Met1–Lys238) without the presence of any affinity-purification tags. Crystals of EGFP^D190Δ^ and EGFP^A227Δ^ grew in space groups *P*3_2_21 and *P*2_1_2_1_2_1_, respectively, with both crystal types containing a single molecule per asymmetric unit. The structures were determined to 1.1 and 1.6 Å resolution, respectively, and were refined to *R* and *R*_free_ values of 14.3 and 16% and of 17.5 and 20.2%, respectively (Table 2 ▶). The final refinement statistics and model geometry fall within the expected range for both crystal structures (Table 2 ▶).

**Figure 3:**
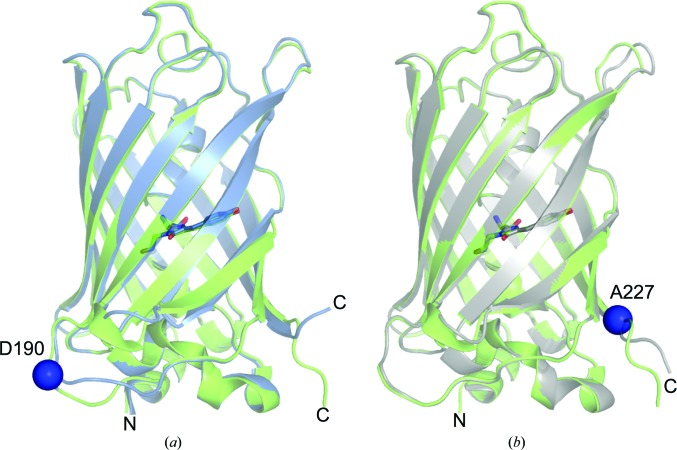
Superposition of EGFP with either (*a*) EGFP^D190Δ^ (blue) or (*b*) EGFP^A227Δ^ (grey). The chromophores are shown in stick representation and the amino acids deleted are shown as blue spheres.

Superpositioning of the structures obtained for EGFP^D190Δ^ and EGFP^A227Δ^ with that of wild-type EGFP shows that the overall structures are very similar (Fig. 3 ▶), with all-atom and backbone r.m.s.d.s of 1.3 and 0.9 Å, respectively (EGFP *versus* EGFP^D190Δ^) or 0.9 and 0.4 Å, respectively (EGFP *versus* EGFP^A227Δ^). This implies that the global structure of EGFP is retained and any structural effects imposed by the single-amino-acid deletions play more subtle roles in local structure rearrangement. This is in line with the general functional features of the variants (Table 1 ▶).

A residue critical to chromophore maturation and spectral properties is Glu222. This acidic residue lies close to the chromophore, and the charged state of the side-chain carboxyl group plays a vital role in defining the charged form of the chromophore in the ground state through the associated hydrogen-bond and charge-transfer network (Tsien, 1998 ▶; van Thor & Sage, 2006 ▶). One of the key mutations in EGFP compared with the original *A. victoria* GFP is the S65T mutation that promotes the red-shifted anionic chromophore form; the molecular mechanism involves changes to the hydrogen-bonding structure around the chromophore so that the neutral form of Glu222 is maintained in the core of the β-barrel. Recent high-resolution structure determination of EGFP has shown that Glu222 exists in two alternate conformations (Arpino, Rizkallah *et al.*, 2012 ▶; Royant & Noirclerc-Savoye, 2011 ▶). Only a single conformation predominated for EGFP^G4Δ^ (Arpino *et al.*, 2014 ▶). As in EGFP, both EGFP^D190Δ^ and EGFP^A227Δ^ exhibited a double conformation for Glu222, as modelling of the Glu222 side chain into two conformations during refinement best satisfied the electron density (Supplementary Fig. S3). The occupancies of confomers Glu222*A* and Glu222*B* were 0.7 and 0.3, respectively, for both EGFP^D190Δ^ and EGFP^A227Δ^, the same as those for EGFP (Arpino, Rizkallah * et al.*, 2012 ▶). The observation of a double conformer for Glu222 in four independently determined high-resolution EGFP crystal structures (the two variants here and the EGFP structures of Arpino, Rizkallah *et al.*, 2012 ▶ and Royant & Noirclerc-Savoye, 2011 ▶) suggest that this is a real structural phenomenon. The absence of a dual conformation for Glu222 in EGFP^G4Δ^ suggests that this deletion mutation has an indirect effect and shifts the conformer population to the dominant *A* conformer. The reasons for and implications of the two conformers of Glu222 are not fully understood. However, it is clear that the alternate conformations alter the hydrogen-bonding and structured water network surrounding the chromophore (Arpino, Rizkallah *et al.*, 2012 ▶). While such variations may not influence the coarse fluorescence properties of EGFP, they may be important in determining a fluorescently viable form of the chromophore, thus affecting parameters such as quantum yield.

### 3.4. Structural impact of Asp190 deletion   

**Figure 4:**
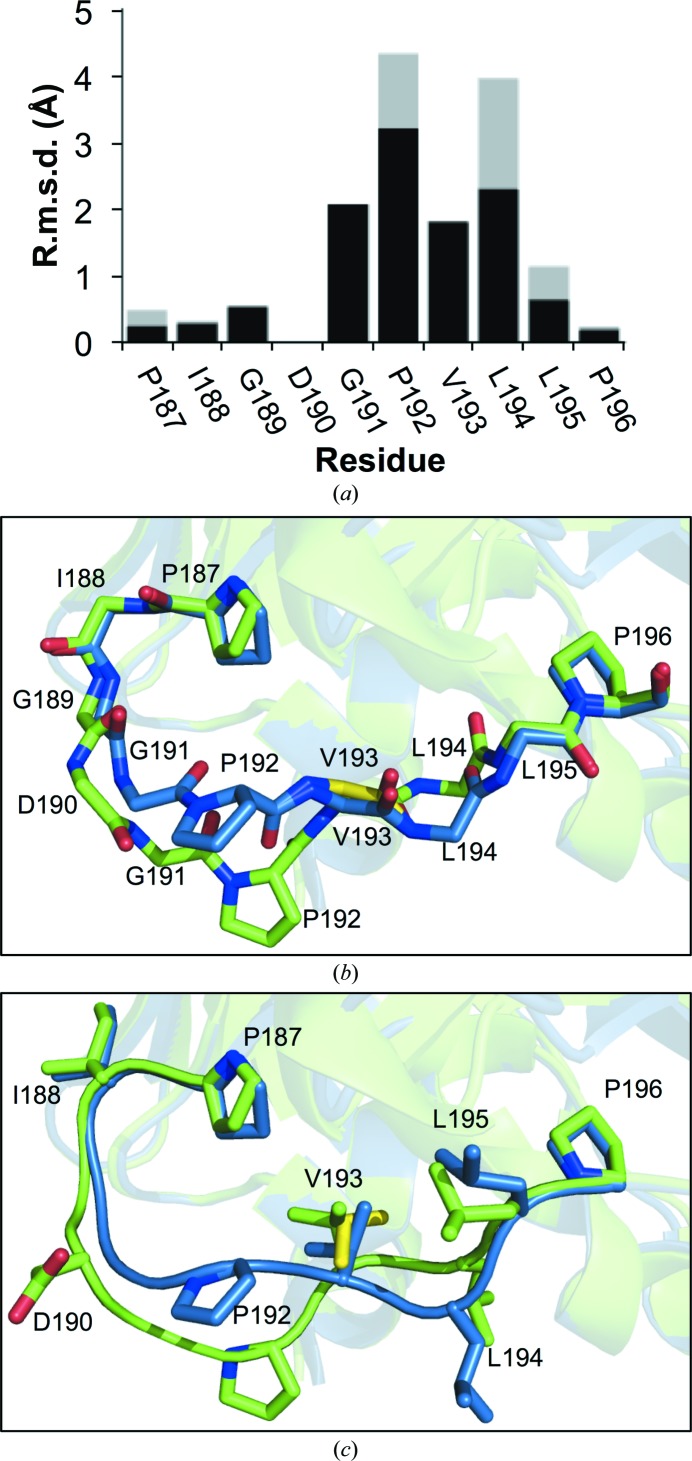
Structural effects of the D190Δ mutation on EGFP. (*a*) R.m.s.d. between EGFP and EGFP^D190Δ^ over the residues immediately before and after Asp190. Backbone atoms and all atoms are coloured black and grey, respectively. (*b*, *c*) Superpositioning of EGFP (green) with EGFP^D190Δ^ (blue) with the backbone (*b*) and the side-chain atoms (*c*) in the loop connecting S9 to S10 displayed. Alternate backbone and side-chain conformations for Val193 in EGFP^D190Δ^ are shown as yellow sticks.

The crystal structure of EGFP^D190Δ^ encompassed residues Lys3–Thr230. In EGFP residue Asp190 is located in a long loop (ten residues) that spans one end of the β-barrel structure linking β-strand S9 to β-strand S10. Thus, the structure of EGFP^D190Δ^ allows us to investigate the structural impact of a deletion in a loop. The backbone trace between the two structures starts to diverge after the deleted residue and does not converge back to the general structure until Leu195 (Fig. 4 ▶). This also results in a significant displacement of the side chains of these residues (Fig. 4 ▶). Therefore, in the present context the main backbone changes are exerted immediately after the deletion and are not propagated either side, with the placement of the flanking β-strands unchanged. The two structures converge around the buried Val193; the residues either side of Val193 exhibit the largest deviations between like residues in EGFP and EGFP^D190Δ^ (Fig. 4 ▶ *a*). Val193 appears to act as a molecular ‘pinch point’ by drawing the loop back to a position closer to that in EGFP (Fig. 4 ▶ *b*). While the r.m.s.d. is still relatively large compared with other residues in the loop, this is predominantly owing to a slight backbone shift; the orientation and thus the registry of the side chain is essentially unchanged (Fig. 4 ▶ *b* and 5 ▶ *c*). This suggests that Val193 may play an important role in acting as an anchor for this loop through maintaining the local hydrophobic interaction network.

**Figure 5:**
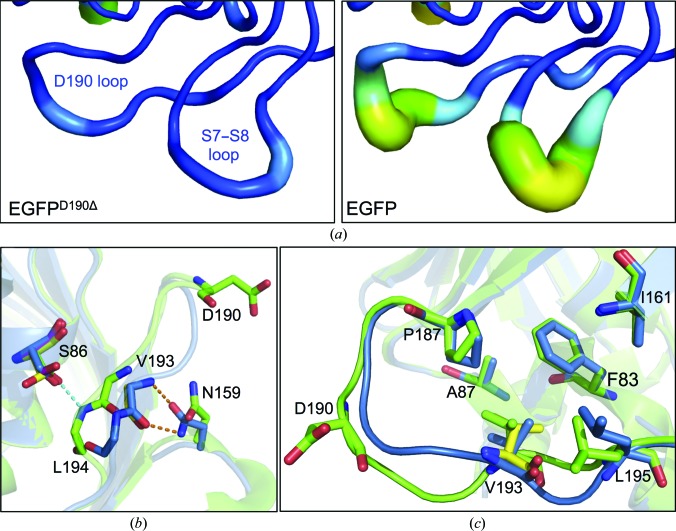
Long-range effects of Asp190 deletion on the EGFP structure. (*a*) Putty diagram illustrating differences in *B* factors for EGFP^D190Δ^ (left) and EGFP (right). Increased *B* factors are shown as increased thickness and a colour transition (blue to orange). (*b*, *c*) The local hydrogen-bond (*b*) and hydrophobic (*c*) networks for EGP (green) and EGFP^D190Δ^ (blue).

Residues within the S9–S10 loop in GFP and its derived variants characteristically have higher *B* factors than the rest of the protein, with the highest values centred on Asp190, indicating potential flexibility/dynamics in this region (Fig. 5 ▶ *a* and Supplementary Fig. S4). The removal of Asp190 significantly lowered the *B* factors, implying that the loop is more structured and less flexible (Fig. 5 ▶ *a*). Surprisingly, the *B* factors in an adjacent tight turn linking β-strands S7 and S8 are also significantly reduced with respect to the same region in EGFP (Fig. 5 ▶ and Supplementary Fig. S4). The distinct difference in the *B* factors for EGFP^D190Δ^ compared with EGFP and other GFP-related structures confirms this is not a crystallographic artefact or owing to a difference in the resolution (Supplementary Fig. S4). Structural heterogeneity in the S9–S10 loop has also been observed by NMR (Andrews *et al.*, 2007 ▶, 2009 ▶). Decreasing the inherent flexibility in the S9–S10 loop and the adjacent S7–S8 loop does not have any obvious effects on function (Table 1 ▶), but may have consequences on stability or even the folding process in terms of defining the nature of the lid of the β-barrel that locks the structure into the final functional folded state (Andrews *et al.*, 2008 ▶, 2009 ▶).

Apart from the changes in loop dynamics within the vicinity of the D190Δ mutation, further subtle and important structural arrangements occur, including the backbone of the turn linking β-strands S7 and S8 (Figs. 2 ▶ *a* and 5 ▶). Analysis of the residues in the two adjacent loops with reduced *B* factors reveal different potential hydrogen-bond interactions owing to the deletion of residue Asp190 (Fig. 5 ▶ *b*) whilst preserving a hydrophobic interaction network (Fig. 5 ▶ *c*). In EGFP the side-chain hydroxyl group of Ser86 is within hydrogen-bonding distance of the backbone N of Leu194. However, deletion of Asp190 alters the conformation of this loop, repositioning it so that the backbone N and O atoms of Val193 are within hydrogen-bonding distance of the carboxamide side chain of Asn159 in the adjacent tight turn linking β-strands S7 and S8. This results in the linkage of different secondary-structure elements in EGFP^D190Δ^ (S9–S10 loop to S7–S8 tight turn) compared with EGFP (H3 to S9–S10 loop), potentially being the reason for the reduced *B* factors of residues in these secondary-structure elements in EGFP^D190Δ^. The repositioning of the loop on deletion of Asp190 results in the loss of the hydrogen-bond interaction between Ser86 and Leu194 seen in EGFP, allowing the side chain of Ser86 to take on one of three possible conformations in EGFP^D190Δ^ (Fig. 5 ▶ *b*). In turn, Leu194 moves from a partially solvent-exposed environment (SASA = 54.4 Å^2^) to a solvent-exposed environment (SASA = 157.9 Å^2^) (Fig. 5 ▶ *b*). Overall, there is a net gain of one hydrogen bond between the residues in EGFP^D190Δ^ compared with EGFP. Whilst the deletion of Asp190 results in altered polar interactions between adjacent secondary structures, a hydrophobic interaction network is maintained between residues Phe83 and Ala87 in H3, Ile161 in S8 and residues Pro187, Val193 and Leu195 in the S9–S10 loop (Fig. 5 ▶ *c*).

Thus, the deletion of a residue within a loop does not just cause general loop shortening: a whole host of local and long-range interactions are lost and formed so as to accommodate such a change. This includes limited convergence or ‘pinching’ later on in the loop if the side-chain interactions are part of an extended hydrophobic interaction. Loops play an important role in defining molecular events, and altering their length provides new routes to new functional features (Afriat-Jurnou *et al.*, 2012 ▶; de Wildt *et al.*, 1999 ▶; Patzoldt *et al.*, 2006 ▶; Simm *et al.*, 2007 ▶; Wood *et al.*, 2009 ▶; Jones *et al.*, 2000 ▶; Jones & Perham, 2008 ▶). Given that loop modifications affect structure and function, loop remodelling is of significant interest in the protein-engineering field (Afriat-Jurnou *et al.*, 2012 ▶; Hu *et al.*, 2007 ▶; Ochoa-Leyva *et al.*, 2011 ▶; Jones & Barker, 2004 ▶; Jones *et al.*, 2000 ▶; Jones & Perham, 2008 ▶), and thus it is important to understand the details occurring on changing loop length rather than making simple assumptions concerning adjustments to residue side chains. This will in turn inform the design process. Indeed, the GFP scaffold is a promising target for loop engineering for applications ranging from fluorescent ‘affibodies’ (Pavoor *et al.*, 2009 ▶) to calcium sensing (Akerboom *et al.*, 2009 ▶) to novel energy-transfer systems (Arpino, Czapinska *et al.*, 2012 ▶).

### 3.5. Structural impact of Ala227 deletion   

**Figure 6:**
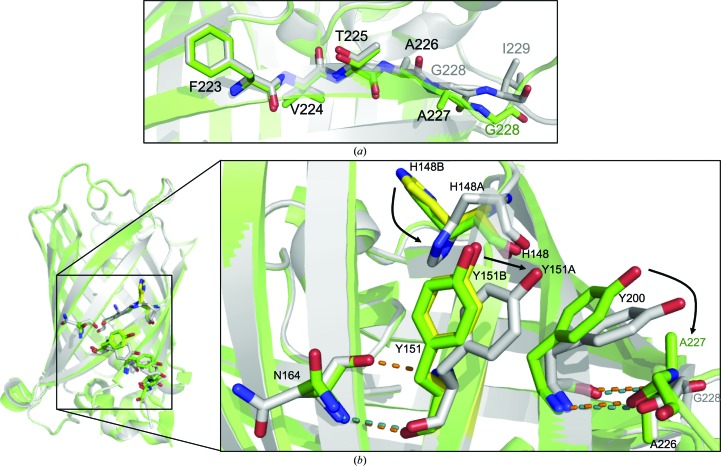
Structural effects of Ala227 deletion on EGFP. (*a*) Superimposition of residues comprising β-strand S11 in EGFP (green) and EGFP^A227Δ^ (grey). (*b*) Changes in long-range interactions. Alternate conformations for His148 and Tyr151 in EGFP^A227Δ^ are shown as yellow sticks.

Ala227 resides at the C-terminus of the final β-strand (S11) that comprises the core β-barrel (Figs. 2 ▶ *b* and 6 ▶ *a*). Residue removal close to the end of β-strands constituted one of the major class of tolerated deletion mutations in EGFP (Arpino *et al.*, 2014 ▶). Thus, it is important to understand the structural impact of such a mutation, especially when it imparts beneficial effects on the protein. The crystal structure of EGFP^A227Δ^ provided structural information from residues Gly4 to Leu231. The main chain of the S11 element itself is not disrupted to any extent, with the main divergence occurring after Ile229 (Fig. 6 ▶ *a*), where electron density becomes less reliable and *B* factors increase. Deletion of Ala227 has little impact on the general structure of S11, with residue removal accommodated by another residue contributing to the β-strand and the termini shortening by one residue. Gly228 moves into the position of Ala227 at the end of S11, with concomitant loss of the Ala227 methyl-group side chain. Thus, it could be envisaged that a similar structural mechanism could be employed when the preceding or following structural element is a loop; β-strand integrity is maintained through structural reorganization of a loop, as observed above for EGFP^D190Δ^. Mutations more central to a β-strand may require more drastic side-chain rearrangements within the context of the strand, as proposed by the original model (Fig. 1 ▶), and thus are unlikely to be tolerated as frequently.

The removal of Ala227 has long-range and indirect effects on the EGFP structure beyond that of S11 (Fig. 6 ▶ *b*). The replacement of Ala227 by Gly228 in S11 generates a hole in the local surface structure of EGFP owing to the removal of the Ala227 methyl group, which is filled by Tyr200 in the adjacent β-strand S10 (Fig. 6 ▶ *b*). The additional space allows the tyrosyl group of Tyr200 to stack more tightly against the β-barrel, which in turn affects the packing of the adjacent Tyr151 tyrosyl group in β-strand S7. The result is that both tyrosine residues are now closely associated with the surface of the β-barrel structure. The electron density for Tyr151 suggested that it exists in two main conformations (Fig. 6 ▶ *b* and Supplementary Fig. S5): one conformer (50% occupancy) similar to that of EGFP and a second conformer with the tyrosyl group π-stacking with the tyrosyl group of Tyr200. The alternate conformations for the surface facing Tyr151 and the nearby His148 (Supplementary Fig. S5) suggest that these residues are in conformational flux. It is not uncommon to observe two alternate conformations for His148 (Reddington *et al.*, 2013 ▶), but for Tyr151 it is much less common. Furthermore, alternate conformations have not been seen before for His148 or Tyr151 in the recently determined structures of EGFP (Arpino, Rizkallah *et al.*, 2012 ▶; Royant & Noirclerc-Savoye, 2011 ▶) or the EGFP^G4Δ^ (Arpino *et al.*, 2014 ▶) and EGFP^D190Δ^ variants, suggesting that such conformational flux may be boosted by the presence of the A227Δ mutation.

**Figure 7:**
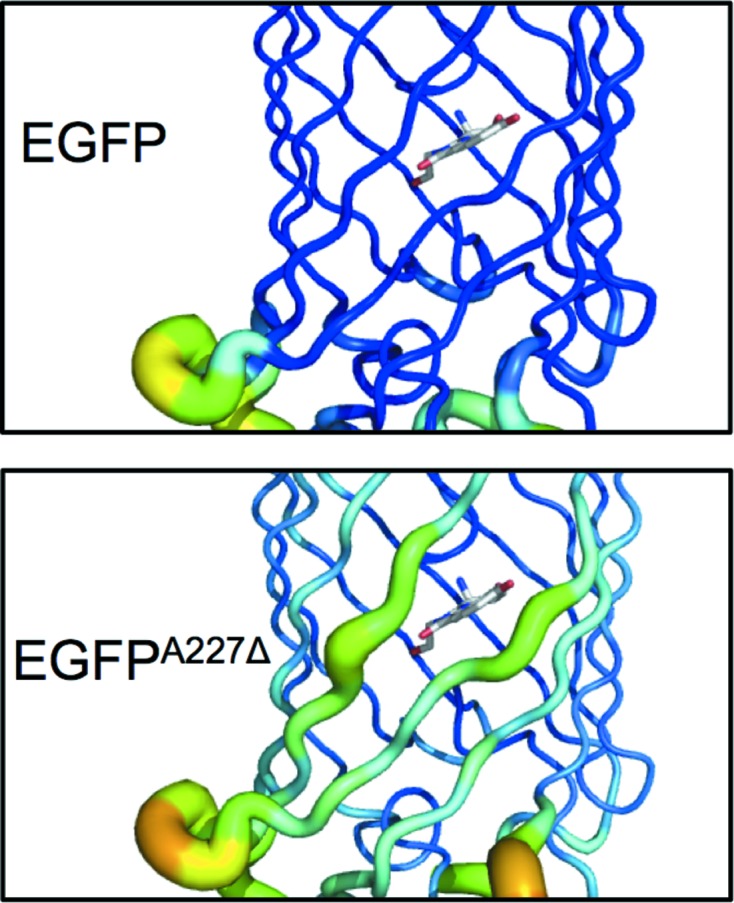
Propagated effect of deleting Ala227 on EGFP dynamics. Putty diagram illustrating the difference in *B* factors for EGFP (top) and EGFP^A227Δ^ (bottom). Increased *B* factors are shown as increased thickness and a colour transition (blue to orange).

It is clear that deletion of Ala227 in β-strand S11 causes a structural ripple across the surface of EGFP to influence not only the adjacent S10 strand but also the indirectly linked strands S7 and S8 (Figs. 6 ▶ *b* and 7 ▶). There is a slight shift in β-strand 7 away from β-strand 8, with a hydrogen bond between the backbone N atom of Tyr151 and the backbone O atom of Asn164 being lost. As well as the loss of a hydrogen bond, repositioning of Tyr151 shifts β-strand S7 enough to allow the side chain of His148 to exist as two conformers (see above). His148 plays an important role in the stability and dynamics of GFP unfolding (Campanini *et al.*, 2013 ▶; Seifert *et al.*, 2003 ▶) and the hydrogen-bond network surrounding the chromophore (Tsien, 1998 ▶). As a result of residues repositioning around the β-barrel, β-strands S7 and S8 are drawn apart from one another, which in turn appears to affect the stability of β-strands 7, 8 and 10, in agreement with previous findings (Campanini *et al.*, 2013 ▶; Seifert *et al.*, 2003 ▶), and is also evident from significantly increased *B* factors for these structures (Fig. 7 ▶ and Supplementary Fig. S6).

Thus, while β-strand S11 housing the deleted residue does not undergo any significant change in structure, the repositioning of residues to compensate for Ala227 deletion results in significant propagated changes across the surface of EGFP, resulting in significant changes in β-strand placement and dynamics. However, apparent increases in flexibility around β-strands 7, 8 and 10 and the perceived change in stability do not appear to have a detrimental effect on the cellular production of EGFP^A227Δ^, as this is significantly enhanced compared with EGFP (Fig. 2 ▶). The importance of Ala227 may be more significant in the folding of the nascent protein before chromophore maturation, as the two forms (nascent polypeptide or unfolded mature polypeptide) of GFP are known to have different folding routes (Hsu *et al.*, 2009 ▶).

### 3.6. Tolerating a deletion in EGFP: helix *versus* strand *versus* loop   

The original simple model proposed in Fig. 1 ▶ suggests a general if rudimentary idea of how deletion mutations are incorporated into various different secondary elements. However, these simple models and perceptions do not explain the details of the events that occur on deletion of an amino acid. In the case of deletion of Asp190, the loop trajectory as a whole does not change but the exact pathway does, although not in a general or a simple manner. Indeed, the paths of the loops begin to coalesce before diverging again. However, events were restricted to residues following the deletion. More drastic examples of structural changes on residue deletion in a loop exist (Stott *et al.*, 2009 ▶), but even here there was a driving force to retain the overall structure and function of the protein. Deletion of residues within loops, such as the ten-residue loop linking β-strands S9 and S10, need not be viewed in isolation through simply reducing loop length. This is clear through the different impacts that deleting different residues within the same loop have (Arpino *et al.*, 2014 ▶). In the case of the removal of a residue from organized secondary-structure elements, there does appear to be a priority in maintaining the secondary-structure element, with local connecting loops accommodating the length reduction in terms of the main-chain changes (Figs. 6*a*; Arpino *et al.*, 2014 ▶; O’Neil *et al.*, 2000 ▶; Vetter *et al.*, 1996 ▶). However, the ripple effects of these deletions in organized secondary structures can be significant, leading to changes in dynamics (Fig. 7 ▶) and long-range polar interaction networks (Fig. 6 ▶ *b*; Arpino *et al.*, 2014 ▶). In the case of EGFP^G4Δ^, this involved the potential stabilization of a *cis*-proline peptide bond brought about by a registry change in a helix (Arpino *et al.*, 2014 ▶). What is clear is that a deletion in all three elements can generate long-range changes through side-chain rearrangements and ripple effects required to accommodate a deletion. It is these changes that are likely to have the most important influence on protein structure and the potential tolerance of a residue to deletion rather than simple assumptions based on backbone rearrangements. The cooperative nature of the interaction network that comprises the three-dimensional structure of a protein means changes distant from the mutation site can and do occur. The nature of the precise changes depends on the context of the residue deleted, making the proposal of general rules difficult. However, knowing the likely main-chain conformational preference on residue deletion will be of great help in the design and modelling process; it will allow more accurate determination of side-chain placement in initial models as inputs for computational analysis, thus preventing ‘dead-end’ nonrepresentative structures from accumulating.

## 4. Conclusion   

Proteins are remarkably plastic structures that are able to tolerate changes to their backbone. Such plasticity is essential for shaping the modern protein repertoire through both the natural evolutionary process and protein engineering. Understanding how deletion mutations, especially beneficial ones, are propagated at the structural level are important for both areas. However, it is especially pertinent for protein engineering, where retrospective analysis of structures can aid future predictive efforts of not only sites that are likely to be tolerated but also those that are likely to be beneficial. The influence of deletion mutations highlighted here and elsewhere (Arpino *et al.*, 2014 ▶) for EGFP exert their effect through more efficient protein production, which is a consequence of efficient protein folding, a feature common to the majority of proteins. However, deletion mutations are not restricted to affecting folding but can affect functional aspects of a protein (Afriat-Jurnou *et al.*, 2012 ▶; Murphy *et al.*, 2009 ▶; Neuenfeldt *et al.*, 2008 ▶; Simm *et al.*, 2007 ▶). Recent whole-proteome analysis has suggested that InDel mutations are important drivers in protein divergence along the protein-fitness landscape, with substitutions acting as enabling or compensating mutations (Leushkin *et al.*, 2012 ▶; Tóth-Petróczy & Tawfik, 2013 ▶). As evolution has proved to be the most effective protein engineer, combining InDels and substitutions either through experimental directed evolution or computationally driven design may be the way forward for generating new proteins of interest.

## Supplementary Material

PDB reference: EGFP, single-amino-acid deletion variants, 4kag

PDB reference: 4kex

PDF of supporting information for qh5010. DOI: 10.1107/S139900471401267X/qh5010sup1.pdf

## Figures and Tables

**Table 1:** Spectral characteristics of EGFP and EGFP variants

Mutation (*Xn*)	_ex_ † (nm)	_em_ † (nm)	‡ (*M* ^1^cm^1^)	§	Brightness¶ (*M* ^1^cm^1^)	†† (ns)
EGFP	488	511	55000	0.60	33000	2.54 0.04
G4‡‡	487	512	53070	0.59	31300	2.64 0.05
D190	486	510	53430	0.58	30990	2.56 0.05
A227	487	511	51850	0.61	31630	2.44 0.04

† _ex_ and _em_ determined from mean fluorescence spectra.

‡ Extinction coefficient determined from single absorbance measurement.

§ Quantum yield determined from integrated fluorescence emission against a fluorescein standard.

¶ Brightness = extinction coefficient quantum yield.

†† Fluorescence lifetimes are mean values with errors calculated from the standard deviation of three measurements.

‡‡ Values for G4 are also reported elsewhere (Arpino *et al.*, 2014 ▶) but are presented here for comparison.

**Table 2:** Cystallographic statistics Values in parentheses are for the last shell.

Variant	EGFP^D190^	EGFP^A227^
Beamline	I03	I04
Wavelength ()	0.97630	0.97950
Space group	*P*3_2_21	*P*2_1_2_1_2_1_
Unit-cell parameters		
*a* ()	57.1	51.5
*b* ()	57.1	63.1
*c* ()	135.3	65.7
Resolution range ()	21.811.14	51.451.60
Total reflections measured	834263	223019
Unique reflections	91397	28209
Completeness (%)	97.3 (75.1)	98.1 (97.4)
*I*/(*I*)	16.1 (2.2)	15.2 (3.4)
*R*_merge_ † (%)	6.5 (62.9)	9.2 (77.7)
*B*_iso_ from Wilson plot (^2^)	10.5	13.4
Refinement statistics		
Protein atoms (excluding H)	2066	1901
Solvent molecules	303	210
*R* factor‡ (%)	13.9	17.4
*R*_free_ § (%)	15.6	20.2
R.m.s.d., bond lengths ()	0.028	0.020
R.m.s.d., angles ()	2.7	2.1
Ramachandran plot statistics		
Core region (%)	98.0	97.3
Allowed region (%)	2.0	2.7
Additionally allowed region (%)	0	0
Disallowed region (%)	0	0

† *R*_merge_ = 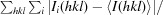 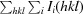.

‡ *R* factor = 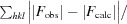 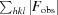.

§ *R*_free_ is calculated from a set of 5% randomly selected reflections that were excluded from refinement.

## Footnotes

1 Supporting information has been deposited in the IUCr electronic archive (Reference: QH5010).
