# Supplementary material for: Structural and dynamic changes associated with beneficial engineered single-amino-acid deletion mutations in enhanced green fluorescent protein
Source: Acta Crystallogr D Biol Crystallogr. 2014 Jul 25;70(Pt 8):2152–62. doi: 10.1107/S139900471401267X (PMC4118826; doi:10.1107/S139900471401267X)
Supplement: Supplementary file 1 [file d-70-02152-sup1.pdf]

SUPPORTING INFORMATION.

Structural and dynamic changes associated with beneficial engineered single amino acid deletion mutations in enhanced Green Fluorescent Protein

James A. J. Arpino[1], Pierre J. Rizkallah[2] & D. Dafydd Jones.[1]

[1] School of Biosciences, Main Building, Park Place, Cardiff University, Cardiff CF10 3AT, UK. [2] School of Medicine, Cardiff University, WHRI, Main Building, Heath Park, Cardiff CF14 4XN, UK.

Supporting Figures.

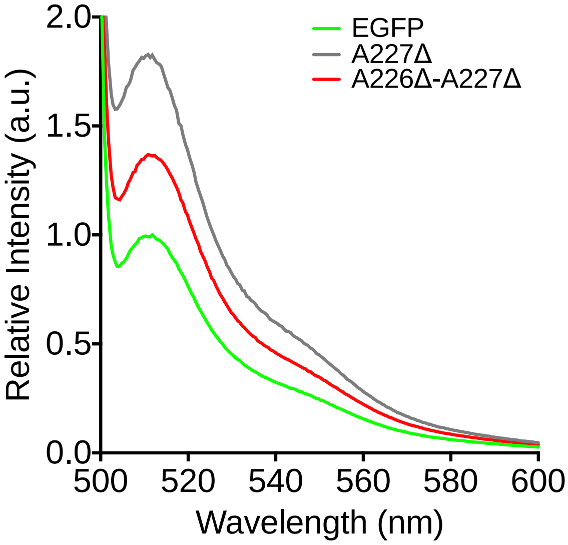

Supporting Fig S1. Whole cell fluorescence spectra. Whole cell fluorescence emission (excited at 488 nm) spectra for cultures grown at 37°C. Cell cultures were standardised to an OD_{600} of 0.1 and spectra normalised to EGFP fluorescence intensity.

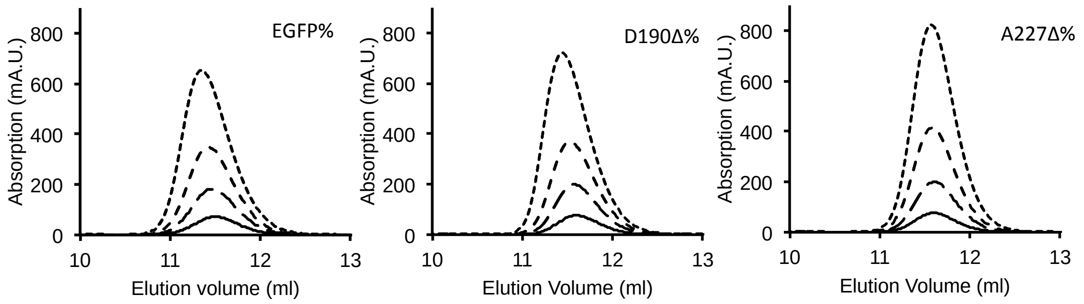

Supporting Figure S2. Size exclusion chromatography of EGFP, $EGFP^{D190\Delta}$ and $EGFP^{A227\Delta}$. The elution profiles of EGFP, $EGFP^{D190\Delta}$ and $EGFP^{A227\Delta}$ at 10 µM (black line), 25 µM (long dash), 50 µM (medium dash) and 100 µM (short dash). The estimated molecular weight based on the peak elution volume are: EGFP ~25.6 kDa (predicted 26.9 kDa); $EGFP^{D190\Delta}$ 24.2 kDa (predicted 26.8 kDa); $EGFP^{A227\Delta}$ 23.4 kDa (predicted 26.9 kDa). Thus all proteins are predominantly monomeric. **Method.** Gel filtration standards (Biorad) were applied to a Superdex™ 75 column (20 ml bed volume, 0.5 ml/min flow rate). As per the manufacturers

guidelines with protein elution monitored at 280 nm. A standard curve was generated from the plot LogMw against K_{av}, where $K_{av} = (V_e\text{-}V_o)/(V_t\text{-}V_o)$, V_e is the elution volume, V_t is the total volume and V_o is the void volume. Protein samples were prepared in Buffer B to final concentrations of 25, 50 or 100 uM and applied to a Superdex™ 75 column with protein elution monitored by absorbance at 488 nm. Elution volumes were determined for each sample and K_{av} values calculated. Using the standard curve estimated molecular weights could be determined for each protein sample.

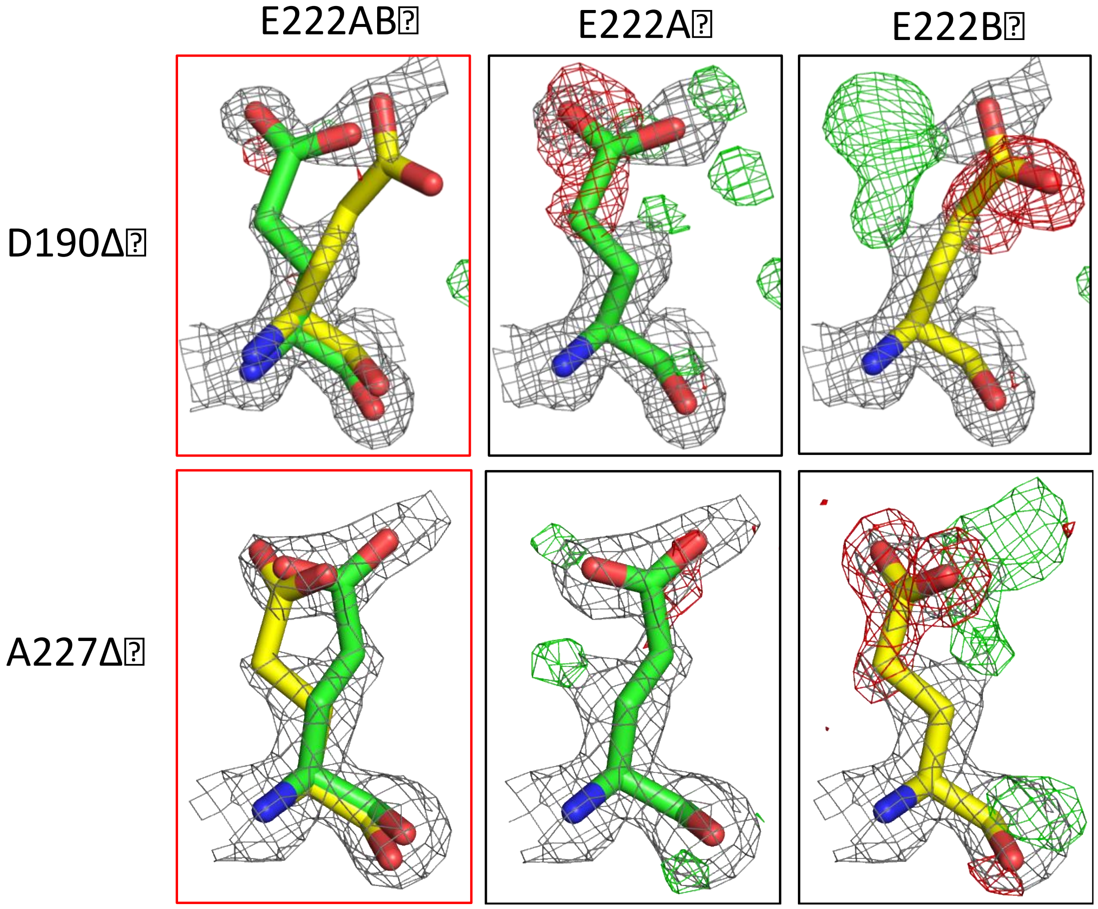

Supporting Figure S3. Rationale behind modelling of E222 as a double conformer in EGFP$^{D190\Delta}$ and EGFP$^{A227\Delta}$. Modelling of residue E222 as either the single conformer A (E222A), the single conformer B (E222B) or as a double conformer (E222AB). The electron density does not fully support the modelling of E222 as a single conformer in either deletion mutant. Occupancy of each form was set to 70% A and 30% B. The model used in final crystal structure refinement is highlighted in the red box (E222AB).

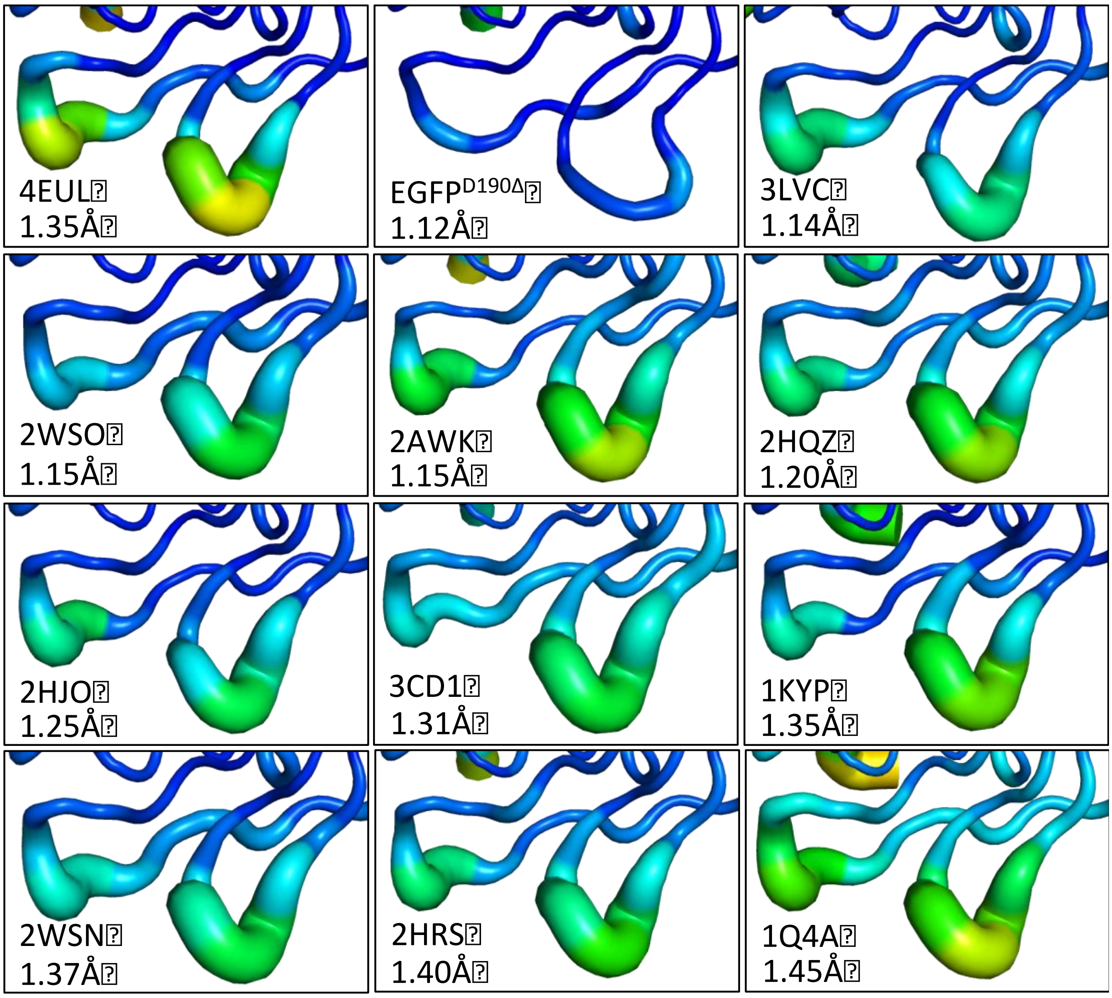

Supporting Figure S4. B-Factor analysis of EGFP, EGFP$^{D190\Delta}$ and other related GFP family members. B-Factor putty representation for EGFP, EGFP$^{D190\Delta}$ and other related GFP structures centred on the loop connecting S9 and S10 and the adjacent tight turn linking S7 and S8. The higher B-factors for these secondary structures in EGP and related family members shows that the significantly lower B-factors observed in EGFP$^{D190\Delta}$ are not a crystallographic artefact and is not due to the high resolution for this structure.

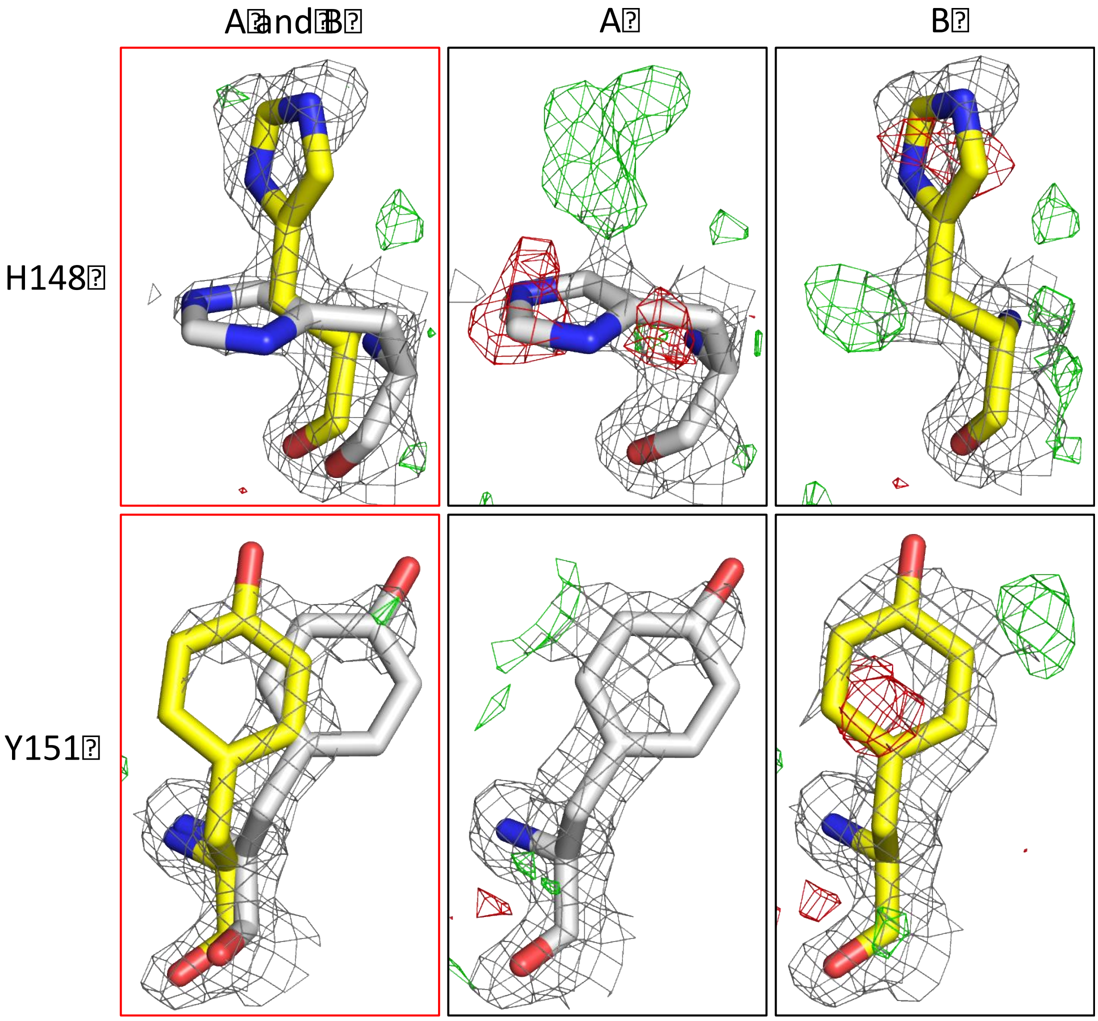

Supporting Figure S5. Rationale behind modelling of H148 and Y151 as double conformers in EGFP$^{A227\Delta}$. Modelling of both H148 and Y151 as either the single conformer A (H148A and Y151A), the single conformer B (H148A and Y151B) or as a double conformer (H148AB and Y151AB). The electron density does not fully support the modelling of H148 or Y151 as either of the single conformers. The model used in final crystal structure refinement is highlighted in the red box with an occupancy of 50% A and 50% B.

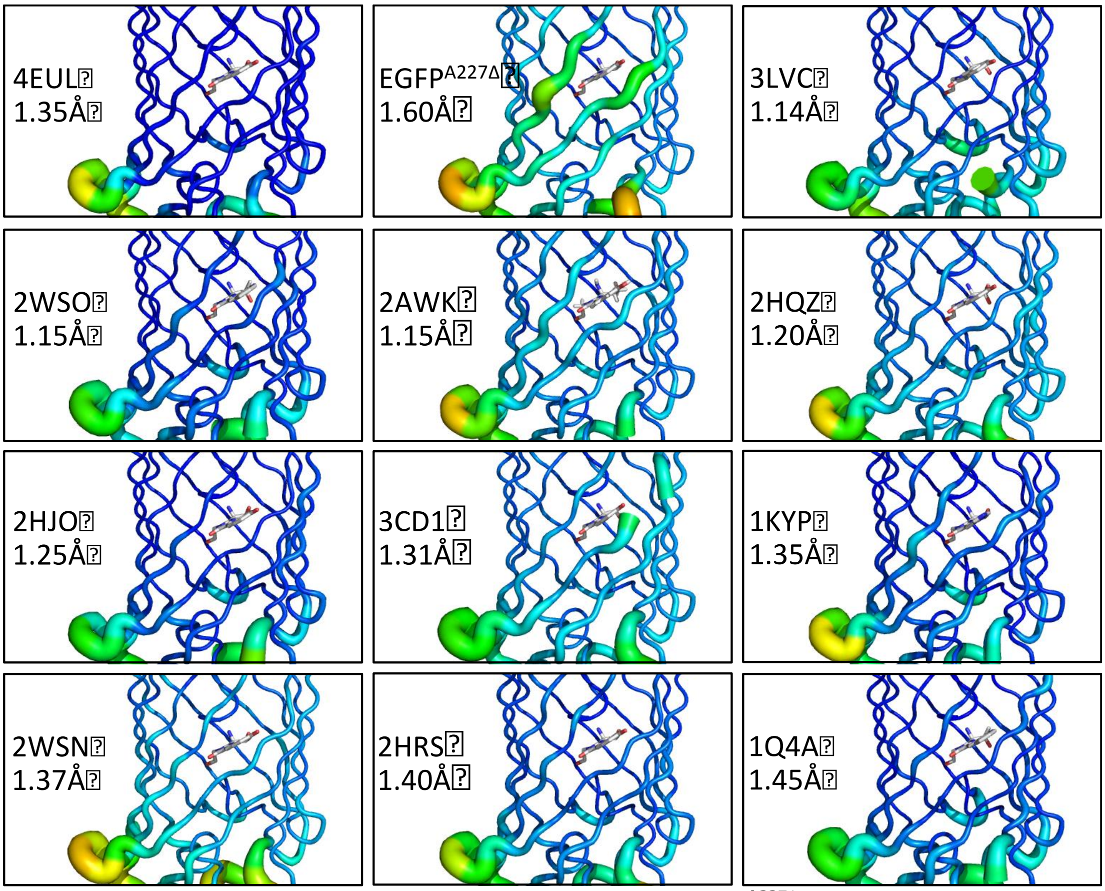

Supporting Figure S6. B-Factor analysis of EGFP , EGFP$^{A227\Delta}$ and other related GFP family members. B-Factor putty representation for EGFP, EGFP$^{D190\Delta}$ and other related GFP structures centred on strands S7, S8, S10 and S11. The higher B-factors for these strands in EGFP$^{A227\Delta}$ in comparison to EGFP and other GFP family members shows that this is not just a crystallographic artefact or due to the resolution.
